# Methods for information-sharing in network meta-analysis: Implications for inference and policy

**DOI:** 10.1017/rsm.2024.17

**Published:** 2025-03-10

**Authors:** Georgios F. Nikolaidis, Beth Woods, Stephen Palmer, Sylwia Bujkiewicz, Marta O. Soares

**Affiliations:** 1 IQVIA, Paddington, London, UK; 2 Centre for Health Economics, University of York, York, UK; 3 Biostatistics Research Group, Department of Population Health Sciences, University of Leicester, Leicester, UK

**Keywords:** aggregate-level network meta-analysis, borrowing strength, evidence extrapolation, information-sharing, multi-parameter evidence synthesis

## Abstract

Limited evidence on relative effectiveness is common in Health Technology Assessment (HTA), often due to sparse evidence on the population of interest or study-design constraints. When evidence directly relating to the policy decision is limited, the evidence base could be extended to incorporate indirectly related evidence. For instance, a sparse evidence base in children could borrow strength from evidence in adults to improve estimation and reduce uncertainty. In HTA, indirect evidence has typically been either disregarded (‘splitting’; no information-sharing) or included without considering any differences (‘lumping’; full information-sharing). However, sophisticated methods that impose moderate degrees of information-sharing have been proposed. We describe and implement multiple information-sharing methods in a case-study evaluating the effectiveness, cost-effectiveness and value of further research of intravenous immunoglobulin for severe sepsis and septic shock. We also provide metrics to determine the degree of information-sharing. Results indicate that method choice can have significant impact. Across information-sharing models, odds ratio estimates ranged between 0.55 and 0.90 and incremental cost-effectiveness ratios between £16,000–52,000 per quality-adjusted life year gained. The need for a future trial also differed by information-sharing model. Heterogeneity in the indirect evidence should also be carefully considered, as it may significantly impact estimates. We conclude that when indirect evidence is relevant to an assessment of effectiveness, the full range of information-sharing methods should be considered. The final selection should be based on a deliberative process that considers not only the plausibility of the methods’ assumptions but also the imposed degree of information-sharing.

## Highlights


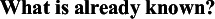

- Various evidence synthesis methods exist for sharing information across different evidence sets.
- Typically, only one information-sharing method is applied, and the impact of this selection is unknown.

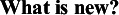

- We describe methods to share information across studies conducted in different populations.
- We implement a range of information-sharing methods to a case-study that borrows strength from paediatric evidence to inform a decision in adults.
- Our findings reveal significant variability in the extent of information-sharing imposed by different methods, highlighting critical implications for inference and policy.

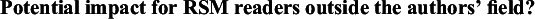

- Information-sharing is pertinent across several areas beyond medical research. The conclusions of this article are generalisable across scientific fields.

## 1 Introduction

Evidence synthesis methods, such as (network) meta-analysis, (N)MA, combine evidence from multiple studies, typically randomised controlled clinical trials (RCTs), on the relative effectiveness between two, or more, health care technologies. Evidence from (N)MA is the cornerstone of health technology assessment (HTA), grounding the clinical and cost-effectiveness assessments supporting clinical decisions and health care policy.

The evidence base for synthesis is typically systematically identified using the PICOS framework, which defines the scope of the literature review (P, population; I, intervention; C, comparator; O, outcome; and S, study-design).^1^ Usually, only evidence that meets the PICOS criteria—that is, *direct evidence*—is retained and synthesised. However, direct evidence may be sparse, biased, or of low internal validity, hindering robust analyses and resulting in highly uncertain estimates.^2^ ^,^ ^3^

To address these challenges, an alternative approach is to expand the evidence base by including indirectly related evidence that retains some relevance to the decision problem. This could entail, for example, using evidence obtained in adults to inform paediatric assessments as considered by the FDA/EMA.^4^ ^,^ ^5^ The term ‘indirect evidence’ refers to evidence whose scope differs in at least one, but not all, of the PICOS domains,^6^ ^,^ ^7^ and generalises from the use of this term in the NMA context which refers only to the Intervention and Comparator domain of PICOS.

In synthesising the two sources of evidence, indirect evidence does not need to be assumed as either perfectly generalisable with the direct evidence (i.e., ‘lumping’) or completely independent from it (i.e., ‘splitting’). Instead, information can be shared to varying degrees between the two sources. Recent work identified and categorised information-sharing methods (ISMs).^6^ ^,^ ^7^ Information-sharing is facilitated by the relationship that the different methods impose between parameter(s) of interest (informed by the direct evidence) and parameter(s) informed by the indirect evidence. Four ‘core’ categories were used^6^ ^,^ ^7^: 1) **functional relationships**, describing deterministic functions amongst the parameters, 2) **exchangeability-based relationships**, assuming that the multiple parameters are independent draws from a common underlying distribution, 3) **prior-based relationships**, where the indirect evidence is incorporated as prior beliefs in a Bayesian framework, and 4) **multivariate relationships** where the parameters are modelled simultaneously, imposing assumptions on their correlation structure.

The aforementioned study also highlighted that existing HTA literature has preferentially used certain core relationships for specific policy problems (e.g., exchangeability-based relationships to facilitate information-sharing between treatments of the same class), without justified reasons for such preferences. Additionally, the study emphasized that different ISMs impose varying degrees of information-sharing, necessitating careful scrutiny. Existing research has not yet explored a broad range of models based on alternative core relationships

This article aims to compare different ISMs and their impact on inference and policy. We apply various ISMs to a case study on Intravenous Immunoglobulin (IVIG),^8^ quantify the strength of information-sharing using a range of alternative metrics, and assess how these models could have enhanced evaluations of clinical effectiveness, cost-effectiveness, and the value of further clinical trials. Additionally, we clarify implementation aspects of the alternative ISMs.

## 2 Description of the case study

Sepsis is an inflammatory response caused by a serious bloodstream infection that can rapidly progress to a life-threatening condition.^9^ Typical SoC treatment includes antibiotics to target the infection, fluids to manage septic shock symptoms, and occasionally albumin (ALB) serum to boost the immune system.^10^

The research question of the original HTA related to the evaluation of the feasibility, cost, and value of information of a multi-center randomized controlled trial of IVIG as an add-on to standard of care (SoC) for adult patients with severe sepsis and septic shock (hereafter referred to as ‘sepsis’).^8^ In this work, we adopt the same research question and further explore how information-sharing might have impacted conclusions relating to relative efficacy, policy making, and further research prioritisation.

### 2.1 Direct evidence of treatment effectiveness in adult patients, and motivation for information-sharing

The evaluation of the case study was based on direct evidence on the relative effectiveness of adjuvant IVIG or IgM-enriched IVIG (IVIGAM) as an add-on to SoC compared to SoC alone in adults, derived from 17 RCTs reporting all-cause mortality.^8^

All 17 RCTs used SoC in their control arm, supplemented with either inactive placebo or albumin placebo. However, both placebo and ALB have disadvantages as control treatments. ALB is similar in appearance to IVIG (colour, transparency, opalescence) but may exert physiological effects that confound inference. Placebo eliminates physiological effects but differs in appearance from IVIG compromising blinding.

These, and similar, issues were explored in detail by Welton et al.,^11^ who identified high levels of statistical heterogeneity, impacting effectiveness and cost-effectiveness analyses. Meta-regression analyses explored the potential for effect modification associated with control type (placebo or ALB), treatment characteristics (e.g., IVIG preparation or treatment duration), and potential sources of bias (e.g., industry sponsorship, sample size, or study quality). Despite extensive analyses, heterogeneity was only partly explained, and the relevance of the underlying sources of heterogeneity remained unclear.

Consequently, multiple evidence synthesis models were proposed for the cost-effectiveness and value of information analysis (Table 1). Conclusions were highly sensitive to the choice of clinical effectiveness model, with the predicted odds ratio (OR) ranging from 0.68 to 1.27, and the value of a new RCT (expected maximum net benefit of sample) ranging from £137 million to £1,011 million.^12^ The authors recommended conducting a high-quality multicenter RCT. However, such a study would be costly, take several years to complete, and, to the best of our knowledge, has not yet been funded. Here, we explore an alternative approach to support decision-making in the adult population by sharing information from other sources of evidence.

**Table 1:** Evidence synthesis models used in the original HTA alongside their predicted odds ratios for all-cause mortality and key cost-effectiveness results

				EVPI per	Optimal
Model	Description	Predicted OR	ICER	patient	sample
1	FE model T3b adjusted for duration of IVIG therapy. Predicted OR for IVIG/IVIGAM vs. albumin for a duration of therapy of three days	0.75 (0.58, 0.96)	£20,850	£1,377	1,900
2	RE model T2 adjusted for Jadad score. Predicted OR for IVIG/IVIGAM vs. albumin/no treatment for a Jadad score of five	0.83 (0.18, 2.13)	£19,968	£4,791	800
3	RE model T3b with no adjustments. Predicted OR for IVIG/IVIGAM vs. albumin.	0.68 (0.16, 1.83)	£16,177	£3,563	1,200
4	RE model T2 adjusted for sample size. Predicted OR for a study with sample size of 339 patients	0.92 (0.23, 2.10)	£28,520	£3,146	900
5	RE model T2 adjusted for sample size. Predicted OR for a study with infinite sample size	1.27 (0.25, 3.17)	Dominated	£2,113	800

*Note*: T2: two treatments considered in the network (IVIG/IVIGAM vs. albumin or no treatment, see Supplementary Material for further details); T3b: three treatments considered in the network (IVIG/IVIGAM vs. albumin vs. no treatment, see Supplementary Material for further details). Abbreviations: EVPI, expected value of perfect information; FE, fixed effecs; IVIG, intravenous immunoglobulin; IVIGAM, IgM-enriched IVIG; OR, odds ratio; RE, random effects.

### 2.2 Broadening the evidence base to include evidence in paediatric patients

In this article, we explore how the body of RCT evidence on IVIG for *paediatric sepsis* patients could support the previous appraisal in adult patients conducted by Soares et al.^8^ Thus, adults remain the primary population of interest, with pediatric evidence used to strengthen relative effect estimates. Notably, a recent study^13^ utilized effectiveness evidence of IVIG in adults to support its potential value in pediatric patients, suggesting that this evidence may be partially transferable between populations. While the aim of this article is primarily methodological it would be crucial to seek clinical expert support for sharing evidence across these sets for decision-making purposes.

We i) updated the previous systematic review of RCTs on the adult population,^8^ and ii) expanded the population criteria to include studies enrolling pediatric patients (see Supplementary Material for full details). We identified 28 studies: 17 enrolling adults (*N* = 2,300 patients, with no new studies since the previous review) and 11 enrolling children (*N* = 4,071 patients). No single study included both children and adults. The largest trial was pediatric, enrolling nearly 3,500 patients.^14^

**Figure 1:**
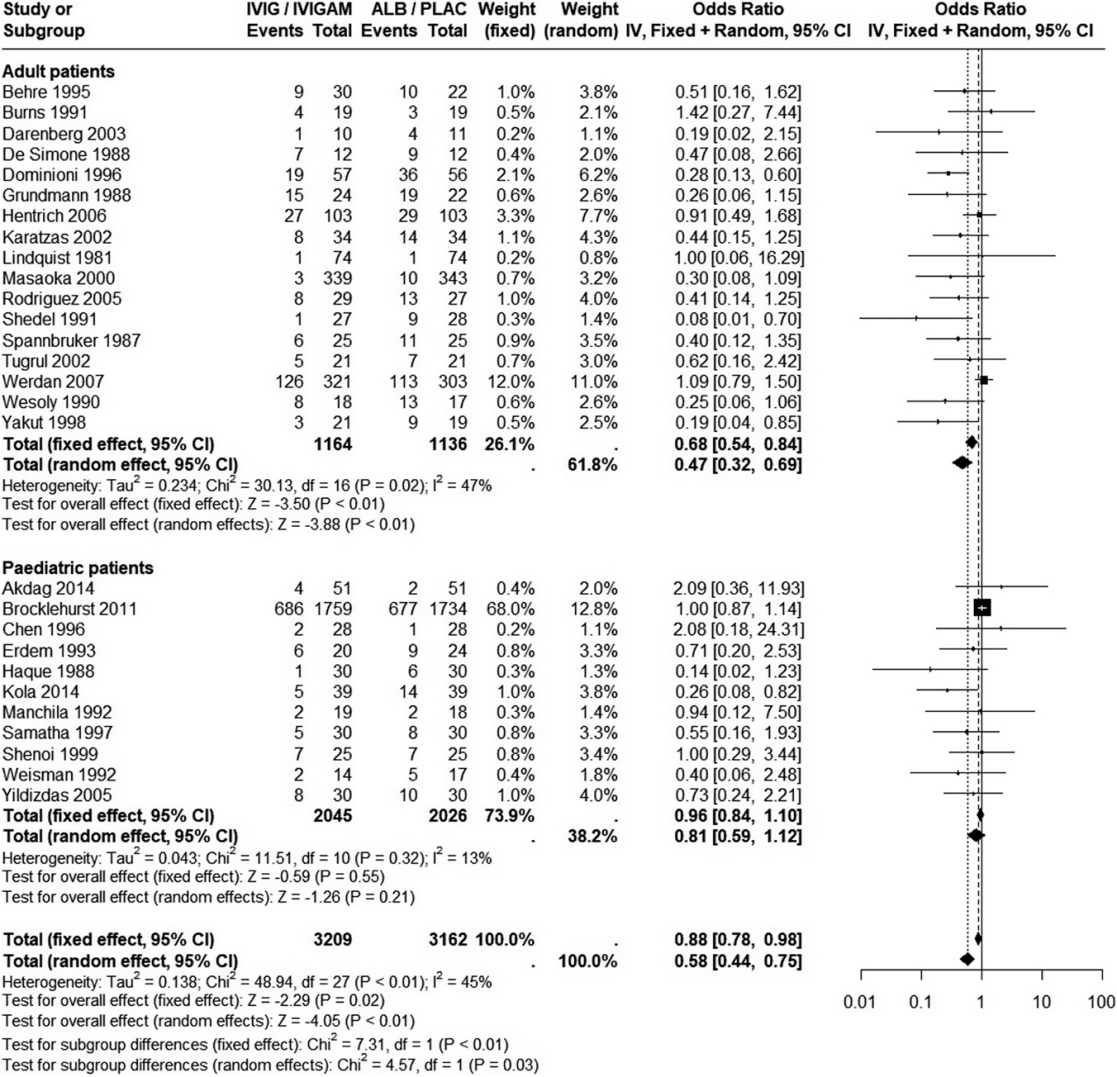
Fixed and random-effects pairwise meta-analyses of all-cause mortality in sepsis, separately within each population and pooled across populations.The evidence base comprises 17 studies in adults (direct evidence) and 11 studies in paediatric patients (indirect evidence). All studies report all-cause mortality. The data are available in the Supplementary Material. Points to the left of the line of no difference favour IVIG/IVIGAM over Albumin/Placebo. The plot was created using the R package ‘*meta*’.

Figure 1 illustrates the direct and indirect evidence base (full data available in Supplementary Material). The pediatric evidence base comprises fewer studies than the adult evidence base, shows significantly less heterogeneity, indicates a treatment effect of lower magnitude, and, unlike the adult evidence, does not produce a statistically significant result (see the random-effects meta-analyses estimates in each evidence set).

## 3 Sharing information on relative effectiveness

In this section, we describe the range of synthesis methods that facilitate information-sharing on relative treatment effect parameters (Section 3.1.1), and the results of their application to our case study (Section 3.1.2).

### 3.1 Evidence synthesis

The different ISMs applied to the case study are summarised in Table 2. Methods were selected by identifying those in Nikolaidis et al.^7^ which could be applied with just one indirect evidence set, and in the absence of studies that incorporate both direct and indirect evidence.

**Table 2:** Summary of ISMs applied in the case-study

ISM (core)	Mathematical relationship	Assumption
Lumping (functional)		RTEs are identical in the direct and the indirect evidence.

Multi-level model (exchangeability-based)		RTEs do not systematically differ between direct and indirect evidence

Commensurate prior (prior-based)		The extent of influence between direct and indirect RTEs is regulated by the variance 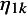 , which is estimated by the model and is therefore adaptive

Informative prior (prior-based)		Indirect evidence is perceived as fully relevant historical data and form an informative prior for the analysis of the indirect evidence

Mixture prior (prior-based)		Indirect evidence is perceived as partially relevant historical data and form an informative prior that is mixed with a vague compenent for the analysis of the indirect evidence. The weight placed on each component is estimated in the model and is therefore adaptive

Power-prior (prior-based)		Indirect evidence is perceived as partially relevant historical data whose likelihood is discounted by a user-specified weight

Abbreviation: RTE, relative treatment effect.

#### 3.1.1 Information-sharing methods

We begin by extending the notation of the standard NMA model^15^ to describe a splitting model, not imposing any information-sharing over the basic parameters, d. We then describe alternative ISMs in the relationship they impose between the basic parameters pertaining to population of direct relevance and the basic parameters pertaining to the population of indirect relevance (i.e., 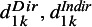 ).


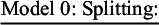
 Consider a set of studies comparing treatment *k* with a reference treatment *b*. Each study, *i*, reports only for one population, 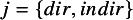 . Therefore, studies can be arranged as 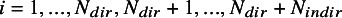 , with 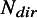 and 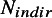 being the total number of studies contributing direct and indirect information, respectively. The synthesis model for a dichotomous outcome (such as all-cause mortality considered here) takes the following form: 
(1)

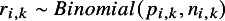



(2)

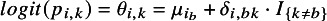



(3)

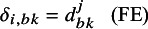



(4)

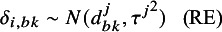



(5)

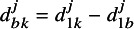



(6)

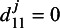

where 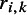 , 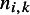 , and 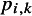 are the number of events, the total number of patients, and the probability of an event in study *i* and arm *k*. 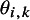 is the linear predictor, 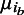 is the study-specific baseline log-odds of the outcome in the reference treatment *b* in trial *i*, and 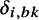 is the study-specific Relative Treatment Effect (RTE) (log odds ratio scale) between the baseline treatment in study *i* and the treatment in arm *k*. Under a FE model, 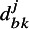 is the population-specific RTE between treatments *b* and *k* (Equation 3). Under a RE model, the population specific parameters are the mean 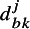 and the variance 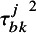 of the Normal distribution describing heterogeneity across the study-specific 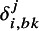 (Equation 4). Between-trial variances are typically assumed common across comparisons (i.e., 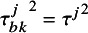 ). Under this splitting model, the basic parameters 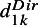 and 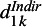 are independent (no information is shared across populations), and are assigned vague prior distributions.

Note that splitting is effectively equivalent to subgroup meta-analysis and is also equivalent to a power-prior with 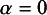 , a mixture prior with a weight of 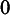 placed on the informative component and approximately equal to a commensurate prior with forced low precision.


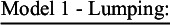
 Lumping is implemented by extending the splitting model to assume 
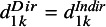
 . Under random-effects, we also lump the heterogeneity parameters ( 
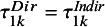
 ) because it is the model most commonly referred to as ‘lumping’ in policy (see Nikolaidis^6^ for more extensive explorations).

Lumping is equivalent to a meta-analysis that does not distinguish between direct and indirect evidence. It is also equivalent to a power-prior with 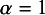 , a mixture prior with a weight of 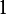 placed on the informative component and approximately equal to a commensurate prior with forced high precision.


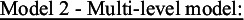
 When the RTEs of the direct and indirect evidence sets are not expected to systematically differ, multi-level models can be applied by extending the splitting-model so that: 
(7)

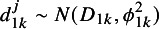

where comparison-specific basic parameters from the different populations are assumed to be normally distributed with a common mean 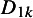 , and a between-populations standard deviation 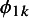 . In the case of two populations, a common heterogeneity parameter across treatment comparisons (i.e., 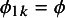 ) is here used to ensure identifiability.


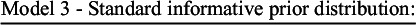
 This comprises of a two-step approach whereby the indirect evidence is first separately analysed using the standard NMA with vague prior distributions, and subsequently the posterior estimates of the first step are used as prior information in a standard NMA model including only the direct evidence: 
(8)

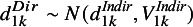

where 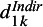 is the posterior mean obtained in step 1, and 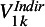 its corresponding variance. Under FE, this model is equivalent to lumping. Under RE, we can use either the posterior mean and its associated uncertainty for 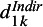 , that is, 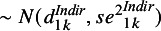 , or its predictive distribution, that is, 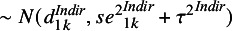 . Here, we choose the latter because we want to ensure that the uncertainty that is due to heterogeneity is appropriately reflected.


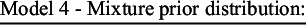
 The mixture prior model is an extension of the informative prior model whereby the posterior estimates of the initial analysis of the indirect evidence are combined with a non-informative prior to form comparison-specific mixture prior distribution for the basic parameters of the direct evidence: 
(9)

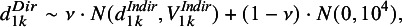

where 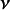 is the weight placed on the informative component, reflecting the plausibility of sharing information between the direct and the indirect evidence sets. Note that, for 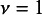 , this is equivalent to the standard informative prior model (model 3), whilst for 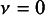 , this is equivalent to splitting (model 0). The parameter 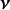 can be assumed, elicited from experts, or estimated within the model by assigning a Beta prior, when the mixture prior comprises only two components, or a Dirichlet prior when it comprises several components. Here, we choose the Beta prior approach, as it has been shown that it offers adaptive information-sharing,^16^ that is, encouraging information-sharing when the direct and indirect sources of evidence are similar, but discourages information when they are ‘in disagreement’.


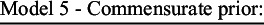
 Commensurate prior models, recently used to synthesise individual- and aggregate-level evidence,^17^ were here adapted to the case of multiple population groups. In this approach, the prior distributions for the basic parameters of the direct evidence are centred around the basic parameters of the indirect evidence and the variance of the prior distributions controls the extent of information-sharing. 
(10)

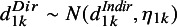



(11)

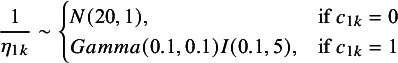



(12)

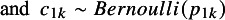

where 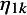 are the comparison-specific variances. Comparison-specific variances imply a different extent of information-sharing (between direct and indirect evidence) across treatment comparisons which may not be plausible, so is it expected that a common variance across treatment comparisons (i.e., 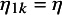 ) would likely be preferred for both simplicity and identifiability purposes. The inverse of the variance parameter (i.e., the precision, 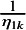 ) is assigned a ‘spike-and-slab’ hyperprior. Such a hyperprior defines two possibilities: one where precision assumes a high value—‘spike’ (defined as a normal distribution centered at 20 and with a low standard deviation of 1)—and hence strong information-sharing is forced, and another where precision assumes a very low value—‘slab’ (defined as a truncated Gamma distribution with shape and rate parameters of 0.1) —imposing minimal information-sharing.^17^ The occurrence of the scenarios is modelled using independent Bernoulli trials (i.e., 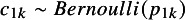 ), with the Bernoulli probability parameter controlling the extent of commensurability, and the strength of information-sharing between direct and indirect evidence. The probability parameter can be fixed to an arbitrary value (e.g., 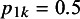 ), or assigned a vague hyper-prior, such as 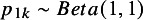 , in order to be estimated within the model. However, in both cases (i.e., 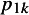 fixed or uncertain), adaptive information-sharing is facilitated, because the choice between the spike and the slab is regulated by 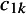 which is estimated in the model. In other words, 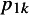 only specifies the value of the Bernoulli prior for 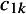 which controls the extent of information-sharing. Therefore, as also highlighted by Hong et al.,^17^ the approach where 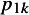 is uncertain is likely to lead to excessive uncertainty. Here, we follow the approach used by Hong et al. (2018) and assume a fixed 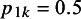 and a spike and slap with hyperparameters as show in Equation (11).


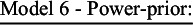
 The power-prior, introduced by Ibrahim et al.^18^ and recently used in the NMA context by Jenkins et al.,^19^ down-weights indirect evidence by raising its likelihood to a power 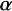 . Under this approach the posterior distribution of the basic parameters of the direct evidence becomes: 
(13)

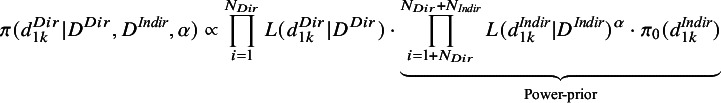

where 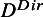 and 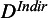 denote the direct and indirect data provided by the corresponding studies. 
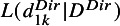
 and 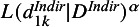 indicate the likelihood of the direct and the indirect evidence and 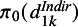 is a vague prior for the basic parameters of the indirect evidence. The 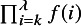 denotes the product of the succeeding expression from study 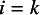 to study 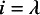 . The parameter 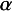 regulates the influence of the indirect evidence: when 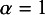 the power-prior is equivalent to lumping, while when 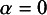 the approach is equivalent to splitting. The value of 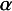 can be arbitrarily defined^20^ or elicited from expert opinion, but it cannot be estimated within the model without model modifications. Here, we used 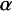 value from 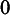 to 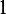 in 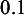 increments.

##### 3.1.1.1 Information-sharing metrics

The level of sharing imposed by each ISM is determined by comparing their posterior estimates with those obtained using the direct evidence only. In what follows, the definition of each metric is described in absolute terms. The three metrics used were:


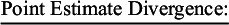
 The point estimate divergence (PED) evaluates the absolute difference in the adult relative effectiveness posterior mean between splitting ( 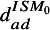 ) and each of the 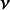 alternative ISMs ( 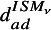 ): 
(14)

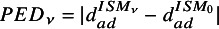

where a larger 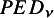 implies a larger difference in the adult point estimate between splitting and ISM 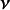 .


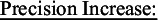
 To capture changes in the standard error of the relative effect estimate obtained by splitting ( 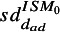 ) and each of the 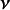 ISMs ( 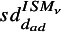 ), we used precision increase (PrI), which was introduced by Jackson et al.^21^ (note that their measure is termed borrowing of strength (BoS). However, multiple measures are used here and this measure is renamed to better reflect the underlying quantity) defined as: 
(15)

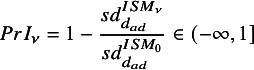

Negative 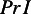 values imply that information-sharing led to increased uncertainty compared to splitting, whilst higher positive 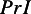 values indicate increasing precision gains.


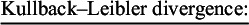
 Finally, we used Kullback–Leibler (KL) divergence^22^ which simultaneously considers changes in the posterior mean and standard deviation. This metric is interpreted as the information conveyed by the probability distribution of 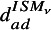 when used to describe another probability distribution 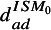 , and is defined as: 
(16)

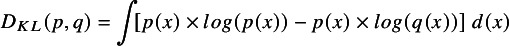

where *p* is the target distribution (here 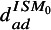 ), and *q* the distribution we use to describe it (here 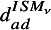 ).

Higher KL values indicate that the amount of information that one distribution conveys about another is reduced and therefore reflect higher information-sharing. However, KL divergence is not symmetrical and does not trade differences in point estimates and uncertainty equally (see illustration in Supplementary Material). Hence, the KL divergence can only be used comparatively across ISMs.

##### 3.1.1.2 Implementation

Synthesis models were implemented in OpenBUGS.^23^ Estimates were obtained from 50,000 iterations (with an additional 20,000 run as burn-in) across three MCMC chains with different starting values. Convergence was checked using the Gelman–Rubin diagnostic—specifically with the multivariate potential scale reduction factor^24^—and visually by assessing the history, chains and autocorrelation. KL divergence was calculated using adaptive quadrature.^6^ Vague prior distributions were used unless otherwise specified.

*Selection of base-models*: Although the original work used five alternative synthesis models in the economic analysis, to facilitate exposition we selected two exemplar models: a fixed effects and a random effects model. The selection of these models was informed by an extended version of the model selection strategy proposed by Welton et al.,^11^ and evaluated the original set of covariates explored to maintain relevance to policy—full details in Supplementary Material. The selected fixed effects model adjusts for the effect of treatment duration in the adult population only—*Base-Model 1*. The estimate deemed relevant for information-sharing was the relative effect of IVIG vs. ALB for treatment duration = 3 days because this duration reflects best practice in adults. To obtain this estimate, the meta-regression model was centered on that covariate value so that the treatment effect coefficient of interest (in this case of IVIG vs. ALB —network T3b) reflects the single (adjusted) estimate of interest for sharing (see Supplementary Material). The random effects model chosen adjusts for Jadad score in both adults and children with a common effect modification coefficient—*Base-Model 2*. The estimate deemed relevant for sharing was the relative effect of IVIG vs. ALB or Placebo (network T2) for Jadad = 5, which reflects the best possible study quality. All ISMs were separately applied to each of the selected estimates from base-models 1 and 2, and the obtained predictions were carried forward to the cost-effectiveness model. Whilst this manuscript explores how a relative effectiveness estimate can be directly informed by extended evidence from a different population, it is important to acknowledge that such an extended evidence-base can also inform heterogeneity explorations and model selection (further examined in the discussion).

#### 3.1.2 Results

**Figure 2:**
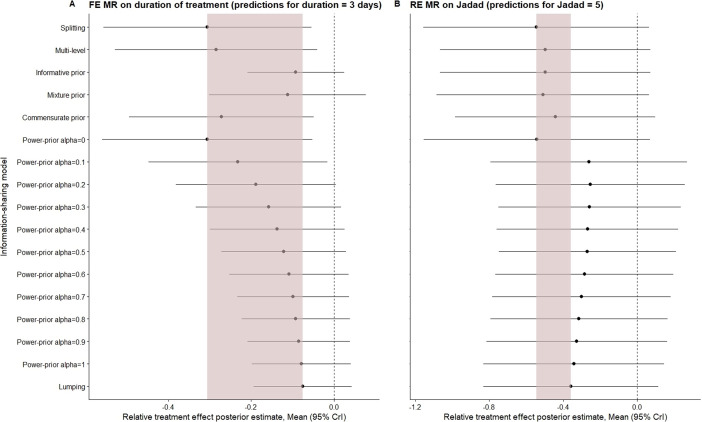
Posterior mean (log odds ratio) estimates for Base-Model 1 and Base-Model 2 across ISMs. Shaded area is defined by the point estimates of the lumping and splitting models. FE, fixed-effects; RE, random-effects; MR, meta-regression; ISM, information-sharing method.

Figure 2 shows a forest plot of the log odds ratio posterior estimates derived from the application of each of the ISMs to both base-models. For both base-models, increasing information-sharing is associated with a shift of the point estimate towards the null effect which is expected since the pooled relative effect in paediatric patients is lower than in adults. The extent of the shift in the point estimate between splitting and lumping is similar across base-models. All FE ISMs yield point estimates that fall within the range defined by the lumping and splitting models (the shaded area in the plot). The standard informative prior model retrieves results equivalent to the lumping model. The multi-level and commensurate prior models yield estimates very close to those of the splitting model. As the value of 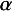 increases from 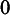 to 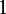 , the power-prior model posterior estimates transition monotonically (though non-linearly) from those of splitting to those of the lumping model. The mixture prior model estimates fall closer to those of lumping.

For Base-Model 2, the point estimates from the power-prior models fall outside the range defined by the lumping and splitting models for a wide range of 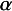 values, showing more extreme values than lumping (towards no effect). This is due to heterogeneity in the indirect evidence, where the large multicenter trial^14^ suggests no effect, contradicting the remaining small studies which suggest a relatively strong effect. Further explorations showed that for 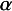 values lower than 0.2, only the likelihood of the large Brocklehurst study gathers weight, with the small studies contributing to the overall effect only at 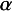 values above 0.2 (see supplementary Material). All other models lie within the spectrum. For the RE analyses, the standard informative prior and the mixture prior models use the predictive distribution to define the prior, resulting in considerably smaller changes in the posterior mean compared to when these approaches are applied to Base-Model 1. The multi-level model results are similar to the informative and mixture prior models. Unlike Base-Model 1, under RE, the commensurate prior deviates considerably from splitting, and aligns more closely with lumping.

**Figure 3:**
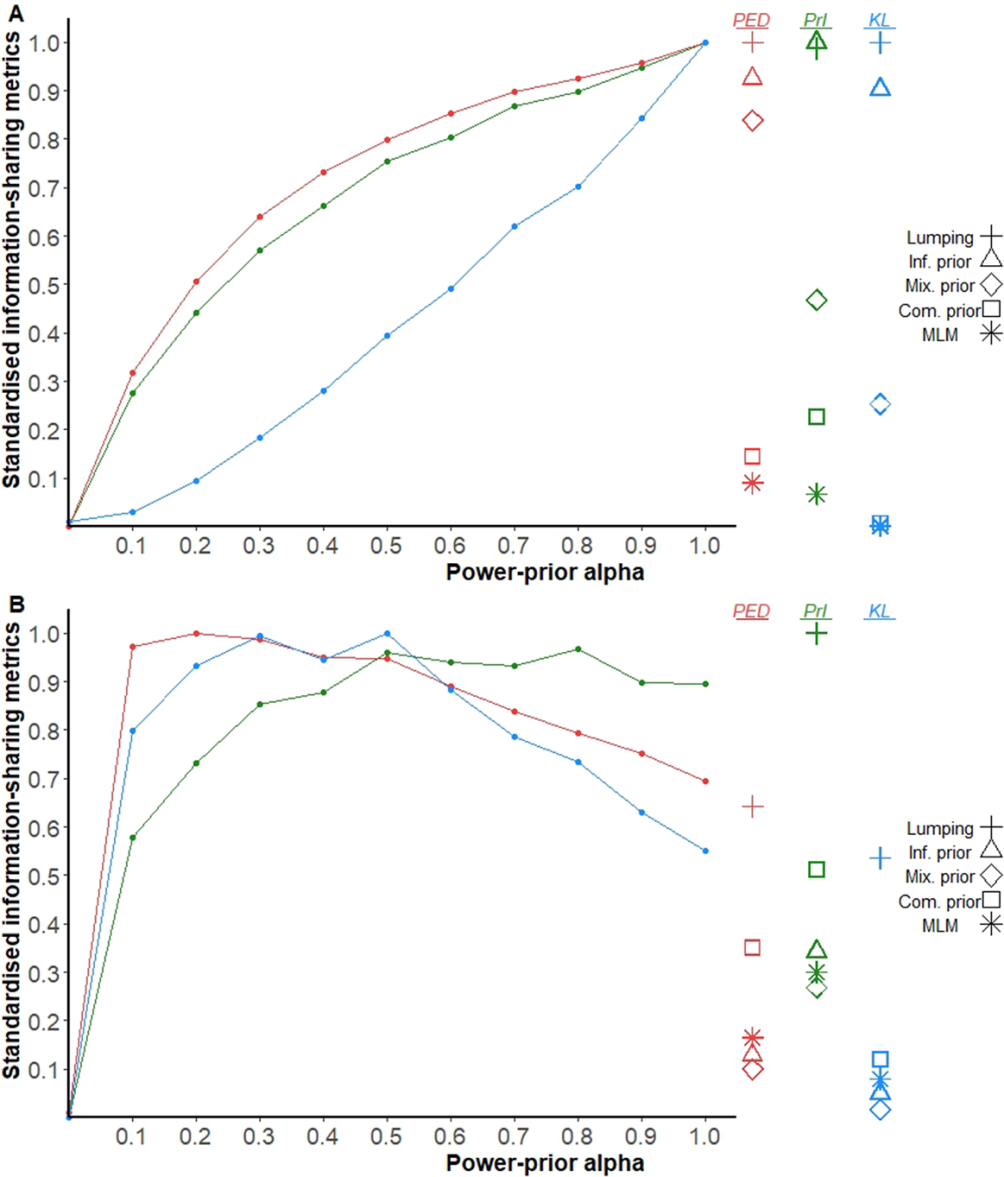
Standardised information-sharing metrics (PED, PrI, KL) of all ISMs for the FE (A) and RE (B) base-models. PED, point estimate divergence; PrI, precision increase; KL, Kullback-Leibler divergence.

Figure 3 shows the information-sharing metrics of all ISMs for Base-Model 1 and Base-Model 2 in panels A and panel B, respectively. The *y*-axis has been standardised to ensure that the metrics range between 0 (no information-sharing) and 1 (full information-sharing). The *x*-axis represents the 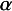 value used in the power-prior model, and hence the lines represent the strength of sharing imposed by the various power-prior models for each information-sharing metric. The remaining ISMs are shown on the right side of the plot, positioned to indicate their imposed strength of sharing for each metric. The graph demonstrates that different ISMs impose varying degrees of information-sharing within each metric, and the strength of sharing imposed by a given ISM can vary across metrics.

### 3.2 Cost-effectiveness

#### 3.2.1 Background and methods

We utilised the decision model developed in the original study, using quality-adjusted life years (QALYs) as the primary outcome and 2009 UK costs from the perspective of the National Health System (NHS). An annual discount rate of 3.5% was applied to both costs and outcomes. Incremental cost-effectiveness ratios (ICERs) were calculated as the ratio of incremental costs to QALYs and compared to common thresholds used for determining the cost-effectiveness of NHS resources.^25^

The model reflects the lifetime prognosis of sepsis to capture the costs and health consequences of sepsis in the absence of IVIG/IVIGAM. There were two distinct model components: first, a short-term decision-tree evaluated the consequences of the initial hospitalisation period following the first sepsis episode. The relative treatment effects obtained from the evidence synthesis were applied to the initial decision tree. Subsequently, conditional on survival in the short term, patients entered a long-term Markov model with two disease states (alive & dead) and annual cycles that captured the long-term consequences for sepsis survivors following the initial hospitalisation.

To understand the potential implications of information-sharing for decision-making, we applied the relative effects estimated by the various ISMs (Section 3.1) to the original cost-effectiveness model. Uncertainty was propagated to the model using probabilistic sensitivity analysis.^26^ We used a cost-effectiveness threshold of £30,000 per QALY gained to determine the probability of a treatment being cost-effective and to evaluate the value of further research.

The value of further research was quantified using value of information methods.^27^ ^,^ ^28^ Four estimates of the value of further research were calculated. The expected value of perfect information (EVPI) estimates the value of resolving all parameter uncertainty relating to the decision problem and represents an upper bound on the value of further research. The expected value of perfect parameter information (EVPPI) estimates the value of resolving uncertainty for individual parameters or groups of parameters. The expected value of sample information (EVSI) quantifies the value of particular research designs using a defined sample size, which are therefore likely to reduce, but not eliminate, uncertainty over particular parameters. Finally, the expected net benefit of sample information (ENBS) was estimated—this subtracts the cost of sampling from the EVSI. These estimates were scaled to the total population expected to benefit from the research over the intervention’s expected lifetime and presented in monetary terms to facilitate comparison with research costs. Further details are available in Soares et al. (Chapter 6, Appendix 5).^8^

#### 3.2.2 Results

The cost effectiveness and value of information results derived using alternative ISMs under both Base-Model 1 and Base-Model 2 are shown in Table 3. The relative KL value from the evidence synthesis models is included as an indication of the degree of information-sharing in relative treatment effect estimates. The results indicate significant variability in adoption and research prioritization decisions under Base-Model 1; under Base-Model 2, the impact is smaller but still noteworthy.

**Table 3:** Model outputs across all applicable ISMs under Base-Model 1 and Base-Model 2 in ascending ICER order. p.EVPPI, p.EVPPI, and max.ENBS in millions pounds sterling (£)

ISM	ICER	p.CE	p.EVPI	p.EVPPI	O.Sample	m.ENBS	KL %
Base-Model 1
Splitting	20,542	0.80	207	75	1,940	41.3	0.0
Power-prior 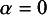	20,539	0.80	206	75	1,940	41.3	0.0
Multi-level	21,392	0.77	226	88	2,150	60	0.1
Commensurate prior	21,954	0.76	220	97	2,250	51.6	0.8
Power-prior 	24,204	0.68	286	162	2,800	118	2.5
Power-prior 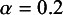	27,723	0.57	373	260	3,400	213	9.8
Power-prior 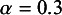	31,381	0.46	366	218	3,400	179	17.8
Power-prior 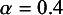	34,863	0.37	252	133	2,900	89.2	28.0
Power-prior 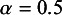	38,176	0.31	175	73	2,600	36.2	38.8
Mixture prior	40,809	0.30	209	112	2,750	70.9	25.5
Power-prior 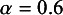	41,603	0.25	128	46	2,300	9.64	49.0
Power-prior 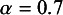	44,871	0.20	90	25	No-RCT	<0	62.0
Informative prior	47,365	0.15	55	14	No-RCT	<0	90.2
Power-prior 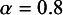	47,393	0.17	71	20	No-RCT	<0	71.4
Power-prior 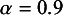	50,907	0.13	51	10	No-RCT	<0	85.2
Power-prior 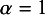	54,116	0.11	39	0	No-RCT	<0	100
Lumping	56,314	0.10	33	0	No-RCT	<0	100
Base-Model 2
Splitting	16,430	0.70	1,091	958	1,040	936	0.0
Power-prior 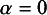	16,458	0.70	1,091	958	1,040	936	0.0
Multi-level	17,131	0.68	1,155	1,029	1,190	1,008	17.6
Commensurate prior	18,066	0.66	1,213	1,096	1,490	1,074	10.9
Mixture prior	19,071	0.69	1,112	983	1,270	961	10.1
Informative prior	19,272	0.68	1,125	988	1,350	976	24.2
Lumping	20,110	0.62	1,273	1,173	1,490	1,150	54.4
Power-prior 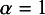	20,826	0.60	1,419	1,327	1,480	1,132	45.4
Power-prior 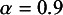	21,428	0.59	1,459	1,370	1,520	1,345	55.3
Power-prior 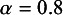	21,983	0.58	1,480	1,394	1,490	1,369	65.9
Power-prior 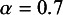	22,737	0.57	1,542	1,460	1,370	1,435	74.7
Power-prior 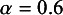	23,484	0.56	1,584	1,505	1,500	1,481	82.8
Power-prior 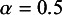	24,460	0.55	1,644	1,569	1,460	1,546	88.4
Power-prior 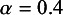	24,472	0.55	1,642	1,566	1,400	1,544	92.9
Power-prior 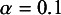	25,200	0.54	1,732	1,659	1,370	1,638	87.7
Power-prior 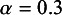	25,424	0.54	1,714	1,642	1,380	1,620	95.8
Power-prior 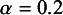	25,536	0.54	1,709	1,637	1,380	1,616	100

*Note*: All estimates, except for ICERs, are calculated using a threshold of £ 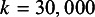 . Informative and mixture prior models under the random-effect base-model use the predictive distribution of the indirect evidence. Abbreviations: ISM, information-sharing method; ICER, incremental cost-effectiveness ratio (in £/QALY); p.CE, probability that IVIG us cost-effective; p.EVPI, population expected value of perfect information; p.EVPPI, population expected value of perfect parameter information for the relative treatment effect; m.ENBS, maximum expected net benefit of sample; O.Sample, the sample size that achieves the maximum ENBS.

Under Base-Model 1, ICERs vary considerably from £20,542 to £55,316 per QALY gained. IVIG could be considered cost-effective if an ISM imposing low information-sharing were used but would have not been considered cost-effective if an ISM imposing strong information-sharing was deemed more appropriate. Under Base-Model 2, ICERs varied less (from £16,430 to £25,530 per QALY gained) as relative effect estimates are larger under Base-Model 2 leading to higher predicted QALYs gains. Reflecting the variation in relative effectiveness estimates (Section 3.1.2), with the power-prior model ICERs follow a non-monotonous relationship with the value of 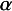 .

The choice of ISM is a key determinant of the value of further research. Under Base-Model 1, ISMs imposing low information-sharing suggest IVIG is cost-effective (ICER < £30,000) with low decision uncertainty (pCE = 0.8). ISMs imposing high information-sharing suggest IVIG is not cost-effective (ICER > £30,000) with similarly low decision uncertainty (pCE = 0.1). Consequently, the resources required for a new RCT are not justified by the benefits of further uncertainty resolution. ISMs with intermediate levels of information-sharing show higher decision uncertainty and value of further research, peaking with the power-prior model at 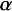 values between 0.2 and 0.3.

Under Base-Model 2, increasing information-sharing leads to higher decision uncertainty and greater population EVPI and EVPPI. All ISMs indicate substantial value in prioritizing a new trial in adults, with EVPPI estimates exceeding £1 billion, which is substantially lower than the cost of a trial (indicatively, a two-arm trial enrolling 1000 patients per arm is assumed to cost around £15 million). The optimal arm sample sizes are more homogeneous, ranging from 1040 to 1520 patients per arm.

## 4 Discussion

This article is the first to compare information-sharing methods and examine their implications for inference and policy. We illustrate those in the context of a case-study where evidence from a related population (paediatrics) is used to strengthen inferences in the target population (adults) for a decision problem. In our case study, under the fixed effect base-model, increasing information-sharing between populations reduced cost-effectiveness and uncertainty, to the extent that IVIG was no longer deemed cost-effective, and further research was not considered worthwhile. Conversely, under the random-effects base-model, the cost-effectiveness of IVIG in adults remained uncertain regardless of the degree of information-sharing, and a large RCT was valuable.

Our findings highlight the wide-ranging impact of different ISMs, particularly under a fixed effect model, and underscore the role of heterogeneity in determining the level of information-sharing. In our case study, fixed effects models with informative priors imposed stronger information-sharing than mixture priors and commensurate priors, while multi-level models imposed only weak information-sharing, especially with only two evidence sets. The power-prior models did not show a linear or monotonic association with any strength-of-sharing metric. Importantly, applying power prior models within random effects models may result in stronger information-sharing than lumping, complicating the interpretation of the power prior 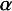 parameter and potentially limiting the ability to elicit such values through structured expert elicitation.

We showed that relative efficacy and cost-effectiveness estimates can be sensitive to the choice of ISMs used in the analyses. Such choice should *not* rely solely on statistical measures like goodness of fit. This is because lack of fit is likely to arise from a conflict between direct and indirect evidence. But, crucially, some conflict may be desirable. For instance, if the indirect evidence is of higher quality than the direct evidence, it may be beneficial to maintain strong information-sharing despite a poorer statistical fit in order to correct for bias. In such scenarios, the indirect evidence provides valuable insights that the direct evidence lacks. Therefore, instead of favouring ISMs that produce better statistical fit, policy and practice should carefully reflect on the possible reasons behind any conflicts between direct and indirect evidence, supported by clinical judgement to determine whether increased information-sharing could lead to better-informed decisions.

The extent of heterogeneity is a crucial determinant of the level of information-sharing. Our study primarily considered the extended evidence-base for information sharing over treatment effect estimates. We re-examined heterogeneity to identify the best fitting fixed- and random-effects base-models in the preparatory stage preceding ISM application. Future research should further explore the value of extended evidence in understanding and explaining heterogeneity, potentially examining methodologies for heterogeneity exploration. In our work, we extended the original approach, grounded on model fitting and selection,^11^ but further research could consider alternative approaches used in practice. We focused on implications of information-sharing for a target estimate of interest obtained from the fixed or random effects base model, without exploration of heterogeneity. Future research could investigate how to integrate heterogeneity exploration and information-sharing in a single stage, assessing the added value of such a joint process and its implications for the chosen analytical approaches and structural assumptions.

In our case study, direct and indirect evidence differed by a study-level population variable (adults/children), with each RCT enrolling patients from only one population. Therefore, methods that are typically used in conventional meta-analysis to explain heterogeneity, such as meta-regression, would not share any information because the effect modification coefficient would ‘absorb’ the effect of the second population. Other conventional methods such as subgroup analyses are also not applicable because, by definition, they do not facilitate any information sharing and separately analyse each population. Hence, the subgroup analysis results are effectively equivalent to splitting.

Other significant areas of uncertainty remain. Further applications and simulation studies could help identify the characteristics of the evidence base that influence the extent of information-sharing imposed by each ISM. Developing more sophisticated metrics to quantify information-sharing and structured expert elicitation methods to transparently inform model choice and specification are also required. Also, it is crucial to develop approaches to determine when information-sharing is relevant, feasible, and worthwhile before extending the evidence base, and across which types of parameters sharing is most appropriate. Finally, to facilitate methodological explorations, we shared information on a single parameter, even though our decision problem involved multiple treatments. Future research should investigate information-sharing across multiple parameters.

Overall, this article shows the range of ISMs that can be applied for sharing information between direct and indirect evidence. ISMs can significantly strengthen inference, and it is therefore important to consider these methods in support of policy-making.

## Supporting information

Nikolaidis et al. supplementary material Nikolaidis et al. supplementary material

## Data Availability

All data and statistical models/programs are provided on the first author’s github https://github.com/NikolaidisGFZ/RSM_Manuscript_Methods_for_information_sharing_in_NMA.git. The data are also provided in the Supplementary Material.

## Supplementary material

The supplementary material for this article can be found at https://doi.org/10.1017/rsm.2024.17.
