# Supplementary material for: Methods for information-sharing in network meta-analysis: Implications for inference and policy
Source: Res Synth Methods. 2025 Mar 10;16(2):291–307. doi: 10.1017/rsm.2024.17 (PMC12527489; doi:10.1017/rsm.2024.17)
Supplement: Nikolaidis et al. supplementary material [file S1759287924000176sup001.pdf]

Methods for information-sharing in network meta-analysis: implications for inference and policy. Supplementary material

Georgios F. Nikolaidis, Beth Woods, Stephen Palmer, Sylwia Bujkiewicz, and Marta O. Soares

1. Systematic literature review methods

The systematic literature review aimed to i) update the review of RCTs on the adult population undertaken within the HTA [1], and ii) expand the evidence base by including studies that enrolled paediatric patients. Therefore, the resulting studies will reflect the current *evidence totality* on both populations. The process comprised of the following steps:

1. The identified studies on adults from Soares et al., 2012 [1] (up to December 2009) along with the identified studies on both adults and paediatric patients from Alejandria et al., 2013 [2] (up to December 2012), which was a systematic review that identified studies in both adults and children, were directly included.
2. The search strategy employed by Soares et al., 2012 [1] to search for adult studies up to December 2009 was used to:
   (a) Identify citations before December 2009 that pertained only to paediatric patients. This was possible because the search strategy did not apply any population criteria, and only excluded studies on non-adult patients during the screening process
   (b) Update the search for both adult and paediatric patients by restricting searches to between 1st January 2010 and 1st August 2018.

The search strategies for MEDLINE and EMBASE are shown in Table 1 and Table 2 respectively.

Table 1: *Search in Ovid MEDLINE(R).*

#	Searches	Results
1	immunoglobulins/	42452
2	immunoglobulin$.tw.	141513
3	ivig.tw.	6165
4	1 or 2 or 3	165054
5	sepsis/	53622
6	sepsis.tw.	83096
7	septic shock/	20856
8	septic shock.tw.	18886
9	septicemia/	53622
10	septicaemia.tw.	6055
11	septicemia.tw.	12227
12	5 or 6 or 7 or 8 or 9 or 10 or 11	139261
13	4 and 12	1778
14	randomized controlled trial.pt.	464602
15	controlled clinical trial.pt.	92507
16	randomized.ab.	407222
17	placebo.ab.	187477
18	drug therapy.fs.	2031668
19	randomly.ab.	288588
20	trial.ab.	423856
21	groups.ab.	1779563
22	14 or 15 or 16 or 17 or 18 or 19 or 20 or 21	4192834
23	exp animals/ not humans.sh.	4475707
24	22 not 23	3617942
25	13 and 24	553

Ovid MEDLINE(R) 1946 to July Week 3 2018, Ovid MEDLINE(R) In-Process & Other Non-Indexed Citations July 31, 2018.

Table 2: *Search in EMBASE.*

#	Searches	Results
1	immunoglobulins/	115815
2	immunoglobulin$.tw.	163890
3	ivig.tw.	14191
4	1 or 2 or 3	235251
5	sepsis/	137769
6	sepsis.tw.	125463
7	septic shock/	44807
8	septic shock.tw.	29733
9	septicemia/	16197
10	septicaemia.tw.	6613
11	septicemia.tw.	13638
12	5 or 6 or 7 or 8 or 9 or 10 or 11	229315
13	4 and 12	5007
14	random.tw.	268899
15	placebo.mp.	407475
16	double-blind.tw.	170545
17	14 or 15 or 16	716448
18	17 and 13	306
19	animals/ not (animals/ and humans/)	1348142
20	18 not 19	306

EMBASE 1980 to 2018 Week 31.

Inclusion Criteria

The inclusion criteria were the same as those used in Soares et. al., 2012 [1], with an important difference being that studies enrolling participants of *any age* were included. In particular:

- Population(s) : Patients of any age with severe sepsis or septic shock
- Intervention(s) : Any preparation of polyclonal IVIG or IVIGAM (i.e., IgM-enriched IVIG)
- Comparator(s) : No treatment (Placebo), Standard of Care (SoC) i.e., antibiotics, or Albumin (ALB) serum
- Outcome(s) : All-cause mortality
- Setting : Critical-care unit
- Study-design : Randomised controlled trials

All studies which investigated the use of IVIG/IVIGAM for *prevention* of sepsis were excluded, along with those studies which had enrolled patients with suspected but unconfirmed sepsis.

Data Extraction

Data were extracted using the template that was developed in Soares et al., 2012 [1]. The following information was extracted:

- Population: Whether the enrolled patient population was paediatric or adult. If paediatric, information on population age (young children, full- or pre-term neonates etc.) was also extracted.
- Intervention: The specific IVIG/IVIGAM product used in the treatment arm, including days of treatment duration, and total dosage (in mg/kg). For control interventions, data extracted predominantly concerned the type of treatment (e.g., no treatment, antibiotics, albumin-serum).
- Outcome: The number of patients enrolled in each arm, along with the number of events (deaths).
- Quality: Allocation concealment, blinding, randomisation, intention-to-treat analysis, missing data; from these, Jadad scores were subsequently calculated [3].
- Other details : Year of publication, setting (e.g., Intensive Case Unit).

2. Analysis data

The full set of data used across all analyses is provided in Table 3.

Table 3: *Direct and indirect data in the IVIG case-study.*

St.ID	t1	r1	n1	t2	r2	n2	pub.year	Jadad	dosage	duration	population	$1/\sqrt{N}$	Study
1	ALB	13	27	IVIGAM	8	29	2005	5	1.75	5	adults	0.19	Rodriguez 2005
2	ALB	29	103	IVIGAM	27	103	2006	3	0.93	3	adults	0.1	Hentrich 2006
3	PLA	14	34	IVIGAM	8	34	2002	2	0.75	3	adults	0.17	Karatzsas 2002
4	PLA	7	21	IVIGAM	5	21	2002	3	0.75	3	adults	0.22	Tugrul 2002
5	ALB	10	22	IVIGAM	9	30	1995	1	0.93	3	adults	0.18	Behre 1995
6	PLA	9	28	IVIGAM	1	27	1991	3	0.855	3	adults	0.19	Shedel 1991
7	PLA	13	17	IVIGAM	8	18	1990	1	0.75	3	adults	0.24	Wesoly 1990
8	PLA	11	25	IVIGAM	6	25	1987	1	0.45	3	adults	0.2	Spannbruker 1987 + Vogel 1987
9	ALB	36	56	IVIG	19	57	1996	3	1	5	adults	0.13	Dominioni 1996
10	ALB	3	19	IVIG	4	19	1991	5	1.2	3	adults	0.23	Burns 1991
11	PLA	9	12	IVIG	7	12	1988	1	1	5	adults	0.29	De Simone 1988
12	ALB	113	303	IVIG	126	321	2007	5	0.9	2	adults	0.06	Werdan 2007
13	PLA	19	22	IVIG	15	24	1988	2	0.5	2	adults	0.2	Grundmann 1988
14	ALB	4	11	IVIG	1	10	2003	5	2	3	adults	0.32	Darenberg 2003
15	PLA	1	74	IVIG	1	74	1981	3	0.45	3	adults	0.12	Lindquist 1981
16	PLA	10	343	IVIG	3	339	2000	3	0.21	3	adults	0.05	Masaoka 2000
17	ALB	9	19	IVIG	3	21	1998	3	1.8	7	adults	0.22	Yakut 1998
18	PLA	1	28	IVIG	2	28	1996	4	0.5	1	children	0.19	Chen 1996
19	PLA	9	24	IVIGAM	6	20	1993	1	0.6	3	children	0.22	Erdem 1993
20	PLA	6	30	IVIGAM	1	30	1988	4	0.5	1	children	0.18	Haque 1988
21	PLA	2	18	IVIG	2	19	1992	3	0.5	1	children	0.23	Mancilla 1992
22	PLA	8	30	IVIGAM	5	30	1997	1	0.6	3	children	0.18	Samatha 1997
23	PLA	7	25	IVIG	7	25	1999	1	0.15	3	children	0.2	Shenoi 1999
24	ALB	5	17	IVIG	2	14	1992	5	0.5	1	children	0.27	Weisman 1992
25	ALB	677	1734	IVIG	686	1759	2011	5	1	3	children	0.02	Brocklehurst 2011
26	PLA	2	51	IVIGAM	4	51	2014	5	0.75	3	children	0.14	Akdag 2014
27	PLA	14	39	IVIGAM	5	39	2014	5	0.6	3	children	0.16	Kola 2014
28	PLA	10	30	IVIG	8	30	2005	3	2	2	children	0.18	Yildizdas 2005

$r1$, $r2$: number of deaths in the control and treatment arms which are of size $n1$ and $n2$ respectively. Dosage is measured in mg/kg and duration of treatment in days.

3. Heterogeneity re-exploration process

3.1. Methods

In the original HTA ([1]), the authors developed and implemented a step-by-step framework to identify important effect modifiers and select the best-fitting models in an evidence base that comprised only of direct evidence [4]. We extended this framework to also include the indirect evidence from paediatric patients. The proposed process not only explores potential effect modifiers and alternative treatment parametrisations separately within each population, but also identifies if and how indirect evidence might help in explaining the heterogeneity among the direct studies.

The extended heterogeneity exploration framework consists of the following steps:

1. Fit simple FE and RE models without covariates: For every possible treatment parametrisation, fit FE and RE models separately in each population without imposing any information-sharing between direct and indirect evidence[1]. Record population-specific residual deviances and between-studies heterogeneities as well as overall DIC and residual deviance[2]. This step provides an initial understanding of the heterogeneity within each population, the extent to which alternative treatment parametrisations can partly explain heterogeneity, and whether or not the relative effects seem to be similar among the two populations.

2. Add covariates: Subsequently, for each potential effect modifier fit the following four meta-regression models:

   (a) FE with separate, population-specific, effect modification
   (i.e. $logit(p_{i,k}) = \mu_i + d_{k,pop} + \beta_{pop} \times cov$)

   (b) FE with common effect modification across the two populations
   (i.e. $logit(p_{i,k}) = \mu_i + d_{k,pop} + \beta \times cov$)

   (c) RE with separate, population-specific, effect modification
   (i.e. $logit(p_{i,k}) = \mu_i + \delta_{i,k} + \beta_{pop} \times cov$)

   (d) RE with common effect modification across the two populations
   (i.e. $logit(p_{i,k}) = \mu_i + \delta_{i,k} + \beta \times cov$)

   In the equations above, pop indexes the population, k the treatment, and i the study. Note that because no study provides information on both populations, pop is nested

[1] This is achieved by specifying separate parameters for each population. The only quantities that refer to the full evidence base are the Deviance Information Criterion (DIC) and the overall residual deviance.

[2] That is simply the sum of the population-specific residual deviances.

in i. Study-specific random-effects are assumed to follow treatment- and population-specific normal distributions ($\delta_{i,k} \sim N(d_{k,pop}, \tau_{pop})$). All remaining parameters in the aforementioned models are defined as in the main body of the manuscript and vague priors are applied to all hyperparameters. Finally, it is important to highlight that models (a) and (c) do not impose any information-sharing among the direct and indirect evidence, whilst models (b) and (d) impose some information-sharing because common effect modification is assumed across the two populations. The process is repeated for all treatment parametrisations.

This step allows us to compare the direction and estimated magnitude of the effect modification for each potentially important covariate in the two populations to assess whether it is statistically reasonable to impose a common effect modification coefficient. We can also obtain additional information regarding the optimal treatment parametrisation, and confirm whether the results are consistent with the previous step, and that the inclusion of covariates has not changed the best-fitting treatment parametrisation.

3. Combining covariates: For the identified important effect modifiers and the best performing treatment parametrisation, we can repeat the process of Step 2, using combinations of covariates. If the results of Step 2 suggest that different covariates are important in the two populations, models which use different effect modifiers for each population can be fit i.e. *'hybrid'* models.

For this analysis, four different treatment parametrisations were explored: T2, T3a, T3b and T4 (Figure 1). The five covariates which had been found to explain some of the heterogeneity in Soares et al., 2012 [1] were considered. These were: duration of treatment, Jadad score, $1/\sqrt{N}$ (sample size), Dosage of IVIG/IVIGAM, and year of study publication.

Models were implemented in *WinBUGS* [5], through *R* [6] using the R2WinBUGS package [7]; a Bayesian framework was adopted. Three MCMC chains with different starting values were used for all models, and Gelman-Rubin statistics were used to assess model convergence. For model comparison, deviance information criterion (DIC) and posterior mean residual deviance (D_{res}) were used and defined in accordance with the NICE Technical Support Document 2 [8]. Models were considered to fit significantly worse when differences in DIC were larger than 3 points [9].

Figure 1: *Updated networks graph.*

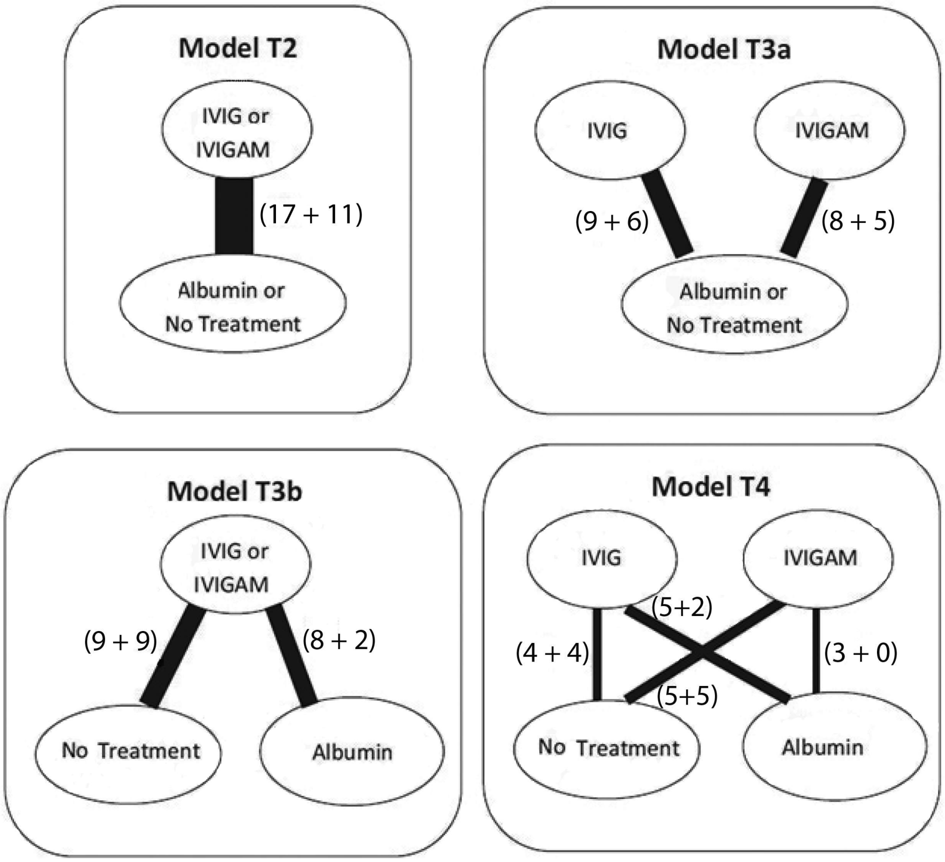

In the parentheses, the first number indicates the number of adult studies providing evidence for the comparison in question, while the second number indicates the number of paediatric studies.

3.2. Results

The results are presented in the same sequence as the steps were described in the methods section. This is because the conclusions of each step feed into the next, until we reach the last step and decide on the final list of base-models. The selected base-models are then the starting point for sharing information among adults and paediatric patients on relative effectiveness.

3.2.1 Step 1 : Fit FE and RE models without any covariates

Table 4 illustrates the results of the application of FE and RE models, without any covariates (null models), in the four treatment parametrisations being assessed.

Table 4: *Results of Step 1 of the re-exploration of heterogeneity.*

Network	Model	τ_{AD}	τ_{PE}	D_{res}	$D_{res_{AD}}$	$D_{res_{PE}}$	DIC
T2	FE	n/a	n/a	77.02	51.43	25.59	303.36
	RE	0.56*	0.47	51.97	30.82	21.14	289.31
T3a	FE	n/a	n/a	71.11	50.12	20.99	299.45
	RE	0.60*	0.35*	51.89	31.23	20.67	289.50
T3b	FE	n/a	n/a	65.61	42.76	22.84	293.91
	RE	0.49*	0.47	52.98	31.57	21.41	290.28
T4	FE	n/a	n/a	65.57	43.58	22.00	295.92
	RE	0.53*	0.46*	52.99	31.90	21.08	291.76

Blue colour indicates a low within-column value, red a high within-column value, and yellow similar within-column values. The asterisk (∗) indicates a significant value at the 95% confidence level. τ refers to the between-studies-heterogeneity and D_{res} to the residual deviance. Subscripts (AD, PE) represent whether a measure only refers to adult or paediatric studies, while when there is no subscript the measure refers to the whole database (adults and paediatric patients).

In all networks, the DIC and residual deviance for RE models are lower than for FE models. The breakdown in residual deviance between the adult and paediatric patients shows that the decrease in residual deviance is mainly driven from the adult evidence (the difference between the paediatric residual deviance across FE and RE models within each network is very small and this is consistent across networks). Across adult studies, heterogeneity (τ_{AD}) is significant regardless of treatment parametrisation, implying that heterogeneity is not adequately explained by the network structure, and therefore covariates will need to be considered.

Across networks, all RE models fit similarly based on both Total Residual Deviance and DIC. However, based on the heterogeneity estimates (τ_{AD}, τ_{PA}), T3b network is the best for adults and T3a for the paediatric studies. However, it should be noted that in

the paediatric network only 2 out of the 11 studies use ALB in the control arm, so when ALB and PLA are separated (as in T3b), only a limited amount of evidence informs the IVIG/IVIGAM *vs* ALB comparison. With regards to T4, DIC and D_{res} are similar to T3b, but heterogeneity estimates are significant for both evidence sets and the model is less parsimonious.

Overall, based on DIC values and given the fact that the primary focus here is on adults, T3b is chosen as the best treatment parametrisation.

3.2.2 Step 2 : Adding covariates

Table 5 shows the results of the meta-regression models, under T3b parametrisation, on a collection of variables which where shown by Welton et al., 2014 [4] to influence the relative effect.

The first feature to notice is that in contrast to the null models, FE models here perform better than RE with the exception of the meta-regression model on Jadad which struggles to explain any heterogeneity. All other models seem to at least partly explain heterogeneity, and improve the fit according to DIC.

The covariate which produces the best performing meta-regression models is duration of treatment, though this improvement seems to be driven only by the adult evidence[3]. This is further supported by the fact that compared to the model that imposes separate effect modification coefficients, when a common effect modification is imposed, the direction of β_{PE} changes and its magnitude becomes very similar to the adult one. As a result of this difference between the two populations, the FE model with population-specific coefficients fits the best in terms of both DIC and residual deviance.

FE meta-regression models on sample size also fit well and marginally better than random-effects. In this case, the magnitude of effect modification is similar among adult and paediatric studies and when a common coefficient is imposed, its estimate becomes more precise (CrI not shown in Table 5). The meta-regression models on year of publication provide a very similar fit with those on sample size, albeit slightly worse in terms of heterogeneity, residual deviance, and DIC.

[3]The fact that duration of treatment does not seem to be an important effect modifier in the paediatric studies may confirm the clinicians' suspicion about this variabe in Soares et al., 2012 [1] where they were unable to intuitively explain the reason that it was the main source of heterogeneity in adults —see Soares et al., 2012 [1] page 38.

Table 5: *Step 2c. Results of meta-regression models on various covariates in network T3b.*

Covariate	Model	τ_{AD}	τ_{PE}	β_{AD}	β_{PE}	D_{res}	$D_{res_{AD}}$	$D_{res_{PE}}$	DIC
NULL	FE	n/a	n/a	-	-	65.61	42.76	22.84	293.91
	RE	0.49*	0.47	-	-	52.98	31.57	21.41	290.28
Duration	FE sep	n/a	n/a	−0.40*	0.54	50.88	27.95	22.94	281.23
	FE com	n/a	n/a	−0.36*	−0.36*	53.25	27.99	25.26	282.58
	RE sep	0.19	0.50	−0.40*	0.54	50.03	28.43	21.60	284.81
	RE com	0.20	0.57	−0.36*	−0.36*	50.50	28.32	22.17	285.26
Jadad	FE sep	n/a	n/a	0.26*	1.97	61.07	38.23	22.85	290.39
	FE com	n/a	n/a	0.26*	0.26*	61.09	38.20	22.88	290.41
	RE sep	0.44‡	0.45	0.18	−3.18	53.86	32.43	21.43	291.07
	RE com	0.43‡	0.47	0.19	0.19	53.84	32.46	21.38	291.10
Sample	FE sep	n/a	n/a	−7.49*	-4.48	55.98	33.09	22.89	286.28
	FE com	n/a	n/a	−6.70*	−6.70*	55.56	32.95	22.61	284.92
	RE sep	0.29	0.50	−6.70*	−4.51	53.22	31.57	21.65	289.36
	RE com	0.27	0.45	−6.22*	−6.22*	52.78	31.47	21.31	287.71
Dosage	FE sep	n/a	n/a	−1.44*	2.06	58.65	35.75	22.90	288.97
	FE com	n/a	n/a	−1.19*	−1.19*	60.61	35.80	24.80	289.95
	RE sep	0.37‡	0.49	−1.25‡	2.02	52.88	31.26	21.62	290.32
	RE com	0.38‡	0.54	−1.01	−1.01	53.05	31.16	21.90	290.31
Year	FE sep	n/a	n/a	0.08*	0.05	57.45	34.65	22.80	287.68
	FE com	n/a	n/a	0.08*	0.08*	56.86	34.47	22.39	286.19
	RE sep	0.31	0.48	0.06	0.06	54.62	32.98	21.63	290.97
	RE com	0.29	0.44	0.07‡	0.07‡	54.08	32.89	21.19	289.24

Blue colour indicates a low value with darkest shading showing the lowest values. The ‡ symbol indicates significance at the 90% confidence level. τ refers to the between-studies-heterogeneity, D_{res} to the residual deviance and β to the meta-regression coefficient of the control variable in question which is modelled in the log-odds ratio scale. Subscripts (AD, PE) represent whether a measure refers to only adult or paediatric studies, while when there is no subscript the measure refers to the whole database (adults and paediatric patients).

Regarding alternative treatment parametrisations, the results of the meta-regression models on all covariates are provided elsewhere [10]. For illustrative purposes though, the models on duration of treatment and on Jadad are provided in Table 6 and Table 7). Just as in Step 1, for the models that account for duration of treatment, T3b is again much better than T2 and T3a, but not significantly better than T4. However, T3b may be preferred over T4 on parsimony grounds. For the models that account for Jadad, T2 fits as well as the other treament parametrisations and should be preferred on parsimony grounds.

Table 6: *Results of meta-regression models on duration for all network parametrisations.*

covariate	Model	τ_{AD}	τ_{PE}	β_{AD}	β_{PE}	D_{res}	$D_{res_{AD}}$	$D_{res_{PE}}$	DIC
T2	FE sep	n/a	n/a	-0.38^*	0.36	61.39	37.11	24.27	289.70
	FE com	n/a	n/a	-0.27^*	-0.27^*	69.01	38.12	30.90	296.34
	RE sep	0.37	0.46	-0.29	0.25	54.01	32.40	21.61	290.33
	RE com	0.43	0.57	-0.18	-0.18	53.28	31.71	21.57	290.52
T3a	FE sep	n/a	n/a	-0.37^*	0.29	58.24	37.57	20.67	288.55
	FE com	n/a	n/a	-0.27^*	-0.27^*	63.53	38.25	25.28	292.85
	RE sep	0.42	0.37	-0.28	0.31	53.14	32.63	20.51	290.34
	RE com	0.49	0.44	-0.14	-0.14	53.55	31.90	21.65	291.56
T3b	FE sep	n/a	n/a	-0.40^*	0.54	50.88	27.95	22.94	281.23
	FE com	n/a	n/a	-0.36^*	-0.36^*	53.25	27.99	25.26	282.58
	RE sep	0.19	0.50	-0.40^*	0.54	50.03	28.43	21.60	284.81
	RE com	0.20	0.57	-0.36^*	-0.36^*	50.50	28.32	22.17	285.26
T4	FE sep	n/a	n/a	-0.41^*	0.54	50.88	28.80	22.08	283.23
	FE com	n/a	n/a	-0.36^*	-0.36^*	53.29	28.85	24.44	284.61
	RE sep	0.21	0.50	-0.40^*	0.54	50.42	29.15	21.27	286.72
	RE com	0.22	0.57	-0.36^*	-0.36^*	51.10	29.14	21.97	287.43

Blue colour indicates a low value with darkest shading showing the lowest values. τ refers to the between-studies-heterogeneity, D_{res} to the residual deviance and β to the meta-regression coefficient of the control variable in question which is modelled in the log-odds ratio scale. Subscripts (AD, PE) represent whether a measure only refers to adult or paediatric studies, while when there is no subscript the measure refers to the whole database (adults and paediatric patients).

Table 7: *Results of Meta-Regression models on Jadad for all network parametrisations.*

covariate	Model	τ_{AD}	τ_{PE}	β_{AD}	β_{PE}	D_{res}	$D_{res_{AD}}$	$D_{res_{PE}}$	DIC
T2	FE sep	n/a	n/a	0.29*	0.09	64.83	39.24	25.59	293.15
	FE com	n/a	n/a	0.20*	0.20*	66.55	39.87	26.68	293.87
	RE sep	0.44	0.55	0.20	0.01	53.78	32.33	21.46	291.51
	RE com	0.47*	0.49	0.12	0.12	53.59	31.53	22.06	290.83
T3a	FE sep	n/a	n/a	0.34*	-0.03	61.59	39.63	21.95	291.92
	FE com	n/a	n/a	0.17*	0.17*	66.80	41.91	24.88	296.11
	RE sep	0.46	0.42	0.23	-0.04	53.86	32.70	21.16	291.90
	RE com	0.54*	0.43	0.08	0.08	53.09	31.63	21.46	291.51
T3b	FE sep	n/a	n/a	0.26*	1.97	61.07	38.23	22.85	290.39
	FE com	n/a	n/a	0.26*	0.26*	61.09	38.20	22.88	290.41
	RE sep	0.44	0.45	0.18	-3.18	53.86	32.43	21.43	291.07
	RE com	0.43	0.47	0.19	0.19	53.84	32.46	21.38	291.10
T4	FE sep	n/a	n/a	0.32*	2.99	60.45	38.43	22.01	291.78
	FE com	n/a	n/a	0.33*	0.33*	60.43	38.42	22.01	291.76
	RE sep	0.46	0.47	0.22	5.57	54.04	32.93	21.10	292.85
	RE com	0.46	0.47	0.23	0.23	53.96	32.91	21.05	292.67

∗ indicates a value that is significant at the 5% level, whilst a ‡ at the 10% level. Blue shading indicates low within-column values.

3.2.3 Step 3 : Combining covariates

The results of Step 2 suggest that duration of treatment is the most important predictor of the relative effect, followed by sample size and year of publication. In this step, these covariates are combined to assess whether the models would fit better and explain a larger part of heterogeneity. The models that combined year with sample size and all three variables together did not converge despite additional efforts such as centering or thinning the MCMC chains. The results of the two remaining combination meta-regression models are shown in Table 8.

The first thing to note is that the effects of sample size, modelled as $1/\sqrt{N}$, and the year of publication are not significant at the 95% level when separate coefficients are assumed in each population. In the first set of models (Duration + Sample), common coefficient models perform slightly better than those with separate coefficients. This may be because the coefficient for duration across paediatric studies has the same sign as in adults, and as a result when a common coefficient is imposed, the residual deviance does not increase and a better fit is observed overall. Heterogeneity in adults is comparable to that estimated with the T3b meta-regression solely on duration (Table 5), indicating that sample size does not explain any additional heterogeneity in adults. However, it does on

Table 8: *Step 3. Meta-regression models with multiple covariates in T3b network.*

Covariate	Model	τ_{AD}	τ_{PE}	$\beta_{1,AD}$	$\beta_{1,PE}$	$\beta_{2,AD}$	$\beta_{2,PE}$	D_{res}	$D_{res_{AD}}$	$D_{res_{PE}}$	DIC
Duration + Sample	FE sep	n/a	n/a	-0.35^*	-0.18	-1.6	-5.7	51.74	28.76	22.98	283.14
	FE com	n/a	n/a	-0.26^*	-0.26^*	$-3.9^‡$	$-3.9^‡$	51.42	28.69	22.72	281.8
	RE sep	0.20	0.48	-0.34^*	-0.04	-2.04	-4.7	50.69	29.12	21.56	286.32
	RE com	0.20	0.47	$-0.27^‡$	$-0.27^‡$	-3.85	-3.85	50.11	28.77	21.34	284.76
Duration + Year	FE sep	n/a	n/a	-0.36^*	6.0	0.015	-0.57	51.82	28.78	23.03	283.26
	FE com	n/a	n/a	-0.27^*	-0.27^*	$0.05^‡$	$0.05^‡$	51.67	28.84	22.82	281.99
	RE sep	0.22	0.50	-0.36^*	-2.5	0.01	0.32	50.63	29.13	21.5	286.3
	RE com	0.21	0.48	-0.29^*	-0.29^*	0.04	0.04	50.78	29.38	21.4	285.7

Blue colour indicates a low value with darkest shading showing the lowest values. τ refers to the between-studies-heterogeneity, D_{res} to the residual deviance, and β_1 and β_2 to the meta-regression coefficient of the first and the second control variable in question which is modelled in the log-odds ratio scale. Subscripts (AD, PE) represent whether a measure only refers to adult or paediatric studies, while when there is no subscript the measure refers to the whole database (adults and paediatric studies).

paediatric studies as can be observed by comparing the paediatric-specific heterogeneity estimates in Table 8 with the corresponding estimates in Table 5, and this leads to lower total and paediatric-specific residual deviance values.

As mentioned above, the model that included both the year of publication and the sample size did not converge. This may indicate that these variables explain the same component of heterogeneity, implying that only one is enough to explain the heterogeneity due to study quality. This hypothesis is further supported by the fact that the last meta-regression models that use the duration of treatment and year of publication perform similarly to the models that use the duration of treatment and the sample size. Finally, given the conclusions from all steps, 'hybrid' models were attempted (Table 9), using different specifications for each evidence set. Note that the first model with FE meta-regression on duration in adults and a FE model without any covariates in paediatric patients provides the best fit amongst all attempted models in this section.

Table 9: *'Hybrid' models that do not use the same covariates in the two populations. T3b network parametrisation.*

covariate	Model ad	Model paed	τ_{AD}	τ_{PE}	β_{AD}	β_{PE}	D_{res}	$D_{res_{AD}}$	$D_{res_{PE}}$	DIC
Duration (adults)	FE M-regr	FE	n/a	n/a	-0.4^*	-	50.82	27.96	22.87	280.1
Duration (adults)	FE M-regr	RE	n/a	0.47	-0.4^*	-	49.34	27.96	21.39	281.7
Dur (ad); Sample (paed)	FE M-regr	FE Mregr	n/a	n/a	-0.4^*	-5	51.28	27.96	23.32	281.9

Blue colour indicates a low value with darkest shading showing the lowest values. τ refers to the between-studies-heterogeneity, D_{res} to the residual deviance and β to the meta-regression coefficient of the control variable in question which is modelled in the log-odds ratio scale. Subscripts (AD, PE) represent whether a measure only refers to adult or paediatric studies, while when there is no subscript the measure refers to the whole database (adults and paediatric studies).

3.2.4 Base model selection

The previous steps illustrated that before imposing any information-sharing, we need to identify the model(s) that best describe the direct and the indirect evidence. Therefore, we investigated heterogeneity in the extended evidence space to find the best fitting models for adults and children. We explored FE and RE models with alternative treatment parametrisations (combining or separating active -IVIG/IVIGAM- and control -ALB/SoC- treatments) as well as meta-regression models using the following effect modifiers: days of treatment duration, Jadad score [3], $1/\sqrt{N}$ (with N being the sample size), dosage of the active treatment, and year of publication. For each covariate we tried models that allowed a different effect modification coefficient for adults and children as well as models with a common coefficient. For covariates exhibiting a significant effect in both populations, models that combined multiple covariates were attempted. Finally, when a covariate was exhibiting a significant effect in one population but not the other, we tried 'hybrid' models that accounted for the effect of the said covariate only in one population.

The model comparison for the base-model selection was based on the DIC in line with [8, 9]. The best fitting model was a 'hybrid' FE model that accounted for the effect of the days of treatment duration in adults but did not consider any covariates for children. This model considered three treatment nodes combining IVIG and IVIGAM but keeping ALB and SoC separate (i.e., T3b). The relative effect of interest there was the ALB *vs.* IVIG/IVIGAM. No random-effects models (with or without covariates) fitted better than the fixed effect model that accounted for the duration of treatment.

However, the aforementioned model adjusted for the effect of the days of treatment duration and as mentioned in [1] clinicians did not find the relationship of this covariate intuitively linked to relative efficacy and suspect that it might be correlated with other influential aspects. Also, since the aim of our paper is primarily methodological, we wanted to understand the impact of information-sharing under RE models. Given that all RE models fitted significantly worse than the aforementioned FE model (i.e., yielded DICs that were higher by at least 5 points), amongst the best fitting RE models, we chose a meta-regression model on Jadad score because it allowed us to predict the relative effect of a study of the best possible quality, and such a model would potentially appeal to decision-makers. Specifically, we chose the RE meta-regression model that used Jadad as a covariate for both adults and children with a common effect modification coefficient. We note that the common effect modification coefficient implies that some information-sharing is already taking place between the two populations. This model combined both active treatments (IVIG/IVIGAM) and control treatments (ALB/SoC). The treatment effect of interest there was hence ALB/SoC *vs.* IVIG/IVIGAM (i.e., T2).

4. Applying information-sharing under meta-regression base-models

Consider the case where the base-models for both evidence sets account for the same covariate X. Then the synthesis model for the direct evidence would take the following form:

$$
\begin{gathered}
r_{i,k} \sim Bin(p_{i,k}, n_{i,k}) \\
logit(p_{i,k}) = \theta_{i,k} = \mu_{i_b} + \delta_{i,bk} + \beta^{Dir} \cdot (X_i - \overline{X_{Dir}}) \\
\delta_{i,bk} \sim N(d_{bk}^{Dir}, \tau^{Dir^2}) \\
d_{bk}^{Dir} = d_{1k}^{Dir} - d_{1b}^{Dir} \\
d_{11}^{Dir} = 0
\end{gathered}
$$

where d_{bk}^{Dir}, d_{1k}^{Dir}, τ^{Dir} are the relative treatment effects, basic parameters, and between-studies heterogeneity specific to the direct evidence. X_i is the study-specific value for covariate X and $\overline{X_{Dir}}$ is the covariate value at which we center. Similarly the synthesis model for the indirect evidence is defined by specifying d_{bk}^{Indir}, d_{1k}^{Indir}, τ^{Indir}, and $\overline{X_{Indir}}$.

The prediction for the relative treatment effect of the direct evidence at a covariate value of, say, $X = 3$ is:

$$
m_{1k}^{Dir}[3] = d_{1k}^{Dir} - \beta_{Dir} \cdot \overline{X_{Dir}} + \beta_{Dir} \cdot 3
$$

which implies that if we choose to center at $\overline{X_{Dir}} = 3$, then two two last components cancel out and

$$
m_{1k}^{Dir}[3] = d_{1k}^{Dir}
$$

Similarly, for the indirect evidence the prediction for the relative effect at $X = 3$, if we choose to also center at $\overline{X_{Indir}} = 3$ is

$$
m_{1k}^{Indir}[3] = d_{1k}^{Indir}
$$

This implies that we can still use the ISMs that are described in the manuscript, without any modifications to accommodate the fact that the relative effect now comprises of two components, even if different β coefficients are used in the two evidence sets, as long as we center both sources' meta-regression models at the covariate value at which we want to relate them.

5. Random-effects extreme information-sharing exploration

In the Jadad RE base-model, for low values of α, the power-prior yields results which share information more strongly than lumping, and as α gets closer to 1, the estimates get closer to lumping. This is attributed to the way that the power-prior operates and to the nature of the indirect evidence.

Specifically, the paediatric evidence includes only one very big study Blocklehurst et al., 2011 (with more than 3000 patients) and 10 relatively small studies (all with less than 100 patients). For very low α values (0 – 0.2), the only study whose likelihood gathers some weight is the big Brocklehurst study, whilst the likelihoods of all the small paediatric studies remain effectively negligible. Therefore, the relative effect of the big Brocklehurst study, which is 0 and therefore suggests that IVIG/IVIGAM is no better than the control, contributes excessively to the overall relative effect and pulls it towards the no effect. For values of $\alpha > 0.2$ the small paediatric studies start becoming non-negligible and their likelihoods acquire adequate weight to be accounted by the random-effects model as small studies. Hence, for values of $\alpha > 0.2$, the small studies start contributing to the overall effect, gradually pulling the relative effect towards the estimate obtained under lumping when $\alpha = 1$.

The above interpretation was tested in a fictitious example that is shown below. In Figure B, the data of the big Brocklehurst study are tweaked so that this study reports a higher relative effect similar to the small paediatric studies. As expected, the result is a monotonous relationship between alpha and the relative effect. In Figure C, the data of the small paediatric studies are tweaked so that they report a very low, close to no effect, relative effect. The result is again a monotonous relationship between alpha and the relative effect in the other direction.

It can therefore be concluded that the reason behind the observed non-monotonous relationship in this case study, leading to some power prior models sharing outside the spectrum defined by lumping and splitting is the nature of the indirect evidence i.e., that the indirect evidence comprises studies that simultaneously considerably differ in their sample size and the relative effect they report.

Figure 2: *Relative treatment effect (IVIG/IVIGAM vs ALB) estimates (y-axis) of re-running power-prior models for different α values (x-axis), for the Jadad RE base-model, under different scenarios for the indirect evidence.*

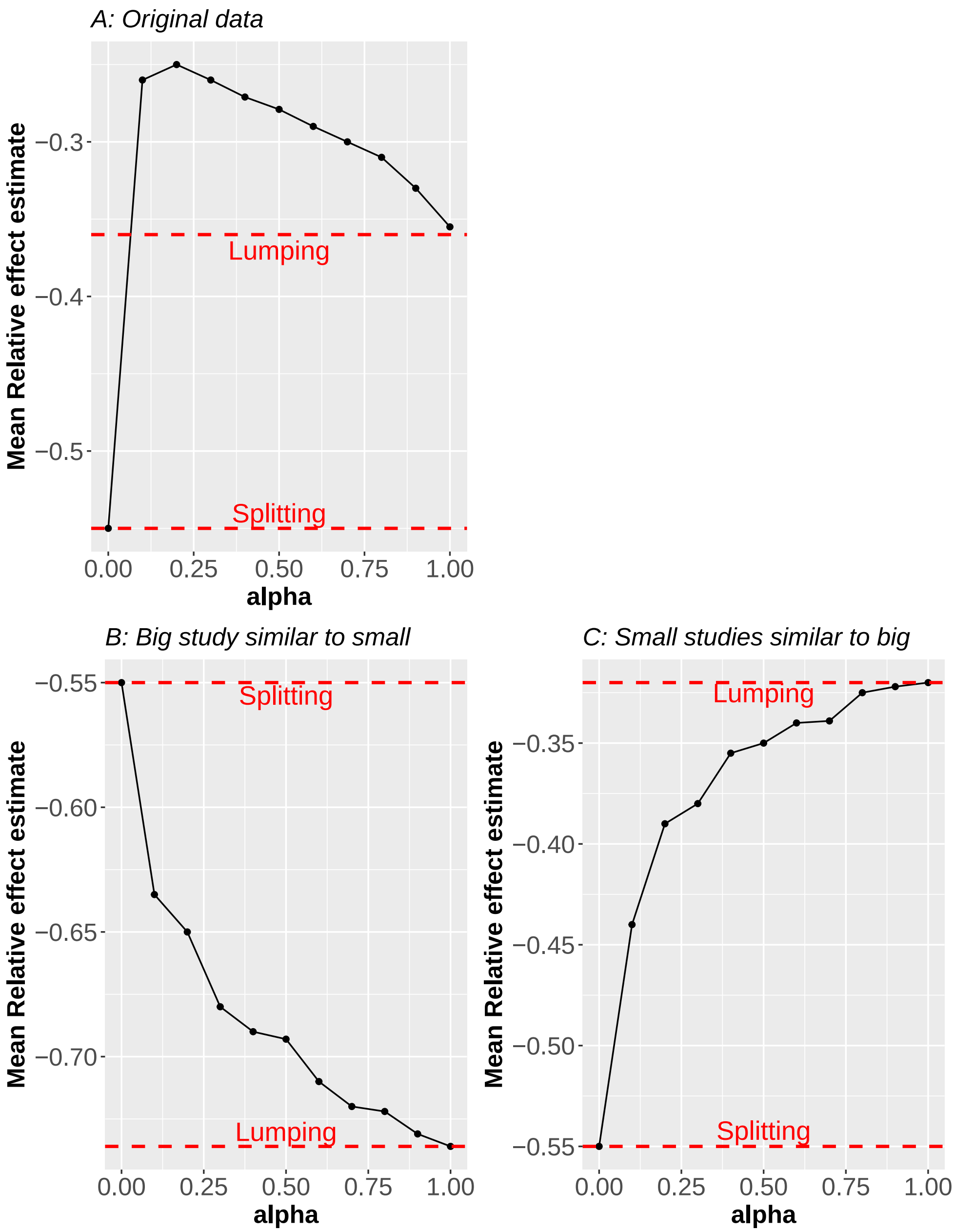

6. Kullback-Leibler divergence illustration

Figure 3 illustrates KL-divergence from a standard normal distribution. The top left figure shows the KL divergence from a standard normal for distributions with $sd = 1$ and means varying between -1 and 1, while the top right graph for distributions with $mean = 0$ and standard deviations varying between 0.4 and 1.6. It is apparent that KL is symmetrical for divergent means, but more sensitive to lower -than the reference distribution- standard deviations. This feature is also revealed in the bottom graph by the non-circular shapes of the oval KL levels lines.

Figure 3: *An illustration of KL divergence from the standard normal distribution.*

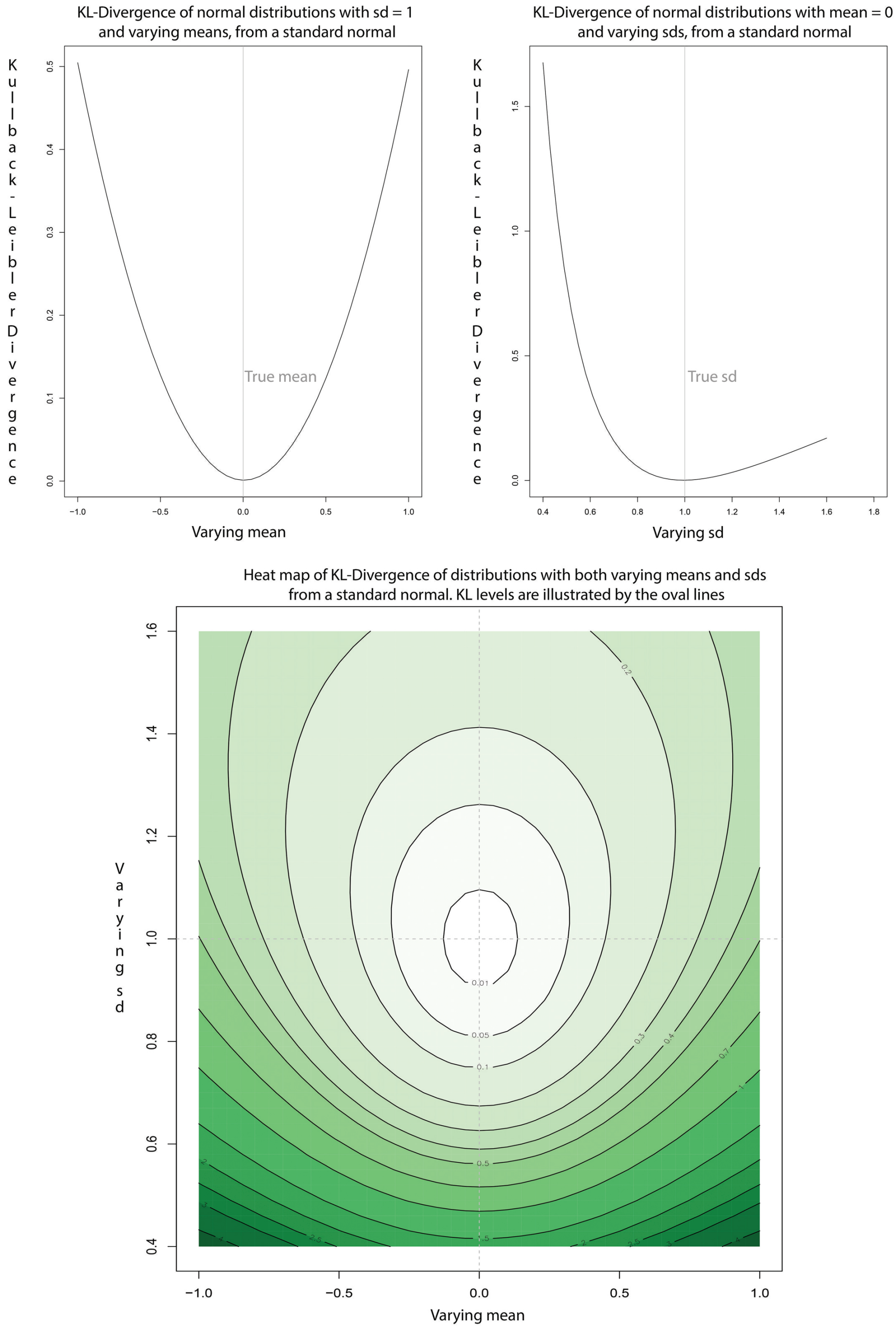

The top left and right graphs show the KL divergence for distribution with varying means and varying standard deviations respectively. The graph in the bottom incorporates both changes simultaneously and reveals the non-symmetrical nature in which KL weights changes in the mean and the standard deviation.

Bibliography

[1] Soares M.O., Welton N.J., Harrison D., Peura P., Shankar H.M.. An evaluation of the feasibility, cost and value of information of a multicentre randomised controlled trial of intravenous immunoglobulin for sepsis (severe sepsis and septic shock): incorporating a systematic review, meta-analysis and value of information analysis. *Health Technology Assessment.* 2012;16.

[2] Alejandria M.M, Marissa M. Intravenous immunoglobulin for treating sepsis, severe sepsis and septic shock *Cochrane Database of Systematic Reviews.* 2013.

[3] Jadad Alejandro R., Moore R.Andrew, Carroll Dawn, et al. Assessing the quality of reports of randomized clinical trials: Is blinding necessary? *Controlled Clinical Trials.* 1996;17:1 - 12.

[4] Welton N.J, Soares M.O., Palmer S.J., et al. Accounting for Heterogeneity in Relative Treatment Effects for Use in Cost-Effectiveness Models and Value-of-Information Analyses. *Medical Decision Making.* 2015.

[5] MRC Biostatistics Unit . The BUGS Project 2010.

[6] R Development Core Team . The R project for statistical computing *The R foundation for statistical computing.* 2010.

[7] Sturtz Sibylle, Uwe Ligges, Gelman Andrew. R2OpenBUGS: A package for Running OpenBUGS from R

[8] Dias S, Welton NJ, Sutton AJ, Ades AE. *NICE DSU Technical Support Document 2: A Generalised Linear Modelling Framework for Pairwise and Network Meta-Analysis of Randomised Controlled Trials*. No. TSD2 in Technical Support Document in Evidence SynthesisNational Institute for Health and Clinical Excellence 2011.

[9] Spiegelhalter D, Best N, Carlin B, Van Der Linde A. Bayesian measures of model complexity and fit *Journal of the Royal Statistical Society: Series B (Statistical Methodology).* 2002.

[10] Nikolaidis G.F.. *Borrowing strength from 'indirect' evidence: methods and policy implications for health technology assessment*. PhD thesis. University of York 2020.
